# Chemoradiotherapy in a Case of Malignant Syringocystadenocarcinoma Papilliferum of Vulva with Locoregional Failure

**DOI:** 10.1155/2015/638294

**Published:** 2015-11-30

**Authors:** Pamidimukkala Bramhananda Rao, Saptarshi Ghosh, Manisha Mohapatra, N. Pramod Philip, P. Ravindra Kumar, Surendra Manam, Pradeep Karra, Vijay Krishna Jasti

**Affiliations:** ^1^Department of Radiotherapy, GSL Cancer Hospital, GSL Medical College, Rajahmundry, Andhra Pradesh 533105, India; ^2^Department of Pathology, GSL Medical College, Rajahmundry, Andhra Pradesh 533296, India; ^3^Department of Radiology, GSL Medical College, Rajahmundry, Andhra Pradesh 533296, India

## Abstract

*Introduction*. Syringocystadenocarcinoma papilliferum (SCACP) is an extremely rare malignant adnexal tumor, which arises from syringocystadenoma papilliferum. To date, less than 30 cases of malignant SCACP have been reported, of which locoregional metastases were found in only four cases.* Case Report*. A 57-year-old female patient who presented to our Oncology Department with a recurrent malignant SCACP of the left labia along with right inguinal lymphadenopathy. Pathological examination confirmed the diagnosis of malignant SCACP with right inguinal lymph node metastases. Due to the fixity of the right inguinal nodes, neoadjuvant chemotherapy was administered with Cisplatin and 5-Fluorouracil for four cycles, following which the primary tumor and the contralateral inguinal nodes regressed completely. Then definitive chemoradiation was delivered with five cycles of weekly Cisplatin and external beam pelvic irradiation up to a dose of 59.4 Gy. Patient is disease-free 11 months after treatment.* Discussion*. We here report the fifth case of malignant SCACP with locoregional metastases. This is the first case of malignant SCACP which has been treated with neoadjuvant chemotherapy followed by concurrent chemoradiation. Although surgery has been used most commonly, chemoradiation may also have a role in the treatment of malignant SCACP, especially in cases of locoregional metastases.

## 1. Introduction

Syringocystadenocarcinoma papilliferum (SCACP) is a very rare entity of cutaneous adnexal tumor, which is listed in the WHO classification of skin tumors as the malignant counterpart of syringocystadenoma papilliferum (SCAP) [1, 2]. SCAP is a benign slow-growing adnexal skin tumor that mostly occurs in the scalp or face [3] and originates from apocrine or eccrine sweat glands [4]. Malignant transformation of preexisting long-standing SCAP into SCACP has been well documented now [5]. Since its first depiction in 1980 by Dissanayake and Salm [6], a total of less than 30 cases of SCACP have been reported till date, of which only four had locoregional metastases [1, 7, 8]. SCACP commonly occurs in the head and neck region, with few of them occurring in other sites [9, 10].

## 2. Case Report

A 57-year-old woman presented to a private practitioner with a swelling in the left labial region in last three years, which was now associated with bleeding, foul smelling discharge, and pruritus since last one month. There were no enlarged inguinal nodes at the time of presentation. She underwent wide local excision alone and was diagnosed with SCACP. Postoperative margin status was negative. Three months postoperatively, she presented to our institutional Oncology OPD with an ulcerative lesion in the left labial region along with right inguinal lymphadenopathy.

**Figure 1:**
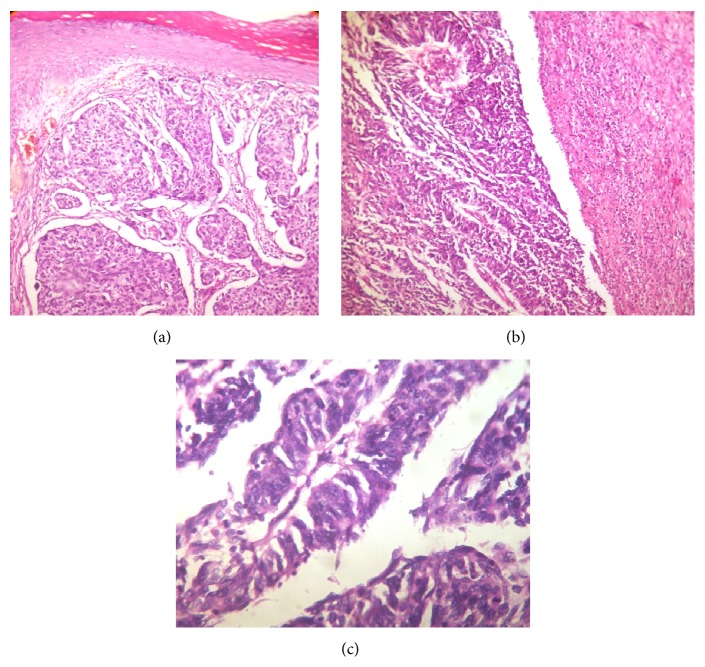
(a) Photomicrograph of the tumor showing epidermis and dermis along with a tumor arranged in tubules, nests, and islands invading into the deeper tissue (H&E, ×100). (b) Photomicrograph showing the tumor arranged in complex papillary fronds having central fibrovascular core; surrounding stroma shows dense lymphocytic infiltration (H&E, ×100). (c) Photomicrograph showing papillae lined by multilayered round to columnar cells showing mild to moderate nuclear atypia, with vesicular chromatin and some showed prominent nucleoli (H&E, ×400).

**Figure 2:**
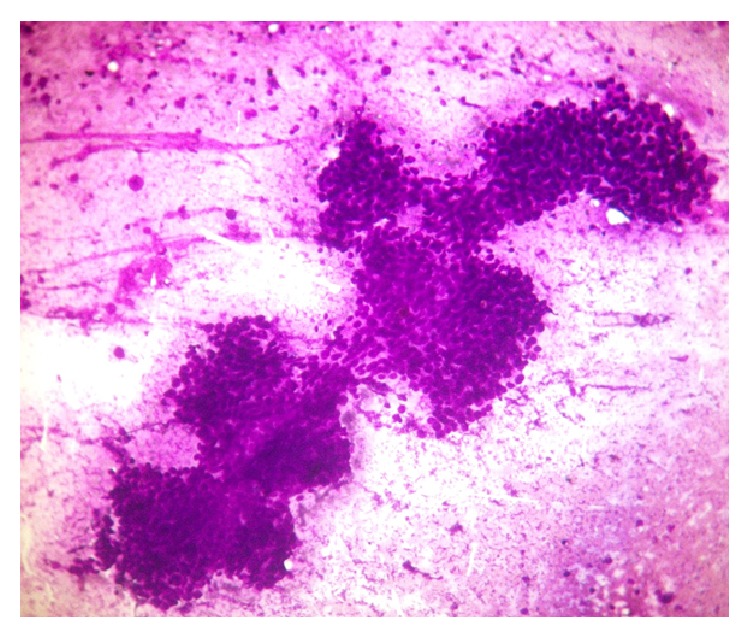
FNA cytosmears from inguinal lymph node showing round to oval tumor cells arranged in papillary structures; background shows many lymphocytes and eosinophilic proteinaceous material (Leishman, ×100).

Histopathological examination of the ulcerative lesion in the left labia showed epidermis comprising of keratinized squamous epithelium and dermis showing a tumor extending from lower epidermis into deep dermis. The tumor was made up of multiple nodules comprising of duct-like structures, some appearing to be cystically dilated, papillary structures having central fibrovascular cores, solid nests, and islands (Figure 1(a)). Dense infiltrate of lymphocytes was seen inside the ducts and papillae and in the surrounding stroma (Figure 1(b)). These ductal and papillary structures were made up of multilayered round to oval to columnar cells having scanty eosinophilic cytoplasm exhibiting moderate nuclear atypia with vesicular chromatin and some showed prominent nucleoli (Figure 1(c)). Histopathological findings favoured the diagnosis of SCACP, residual disease. Fine needle aspiration (FNA) was done from the enlarged firm right inguinal lymph node. The aspirate cytosmears showed abundant cellularity comprising of clusters, nests, papillary structures, and discretely scattered malignant epithelial cells characterized by round to oval cells having scanty eosinophilic cytoplasm exhibiting hyperchromatic or vesicular nuclei with high nuclear-cytoplasmic ratio and some showed prominent nucleoli. Background showed numerous mature and transformed lymphocytes and eosinophilic proteinaceous material (Figure 2). Based on the cytomorphology, a diagnosis of metastatic papillary adenocarcinomatous deposit in right inguinal node possibly from syringocystadenocarcinoma papilliferum of vulva was offered.

**Figure 3:**
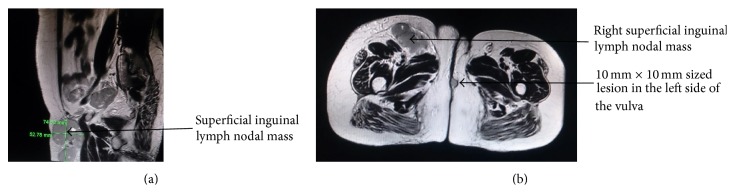
(a) Pretreatment sagittal magnetic resonance (MR) image depicting the superficial inguinal lymph nodal mass measuring 5.3 cm × 7.5 cm in size. (b) Pretreatment axial MR image showing the primary vulvar lesion and the right superficial inguinal lymph nodal mass.

**Figure 4:**
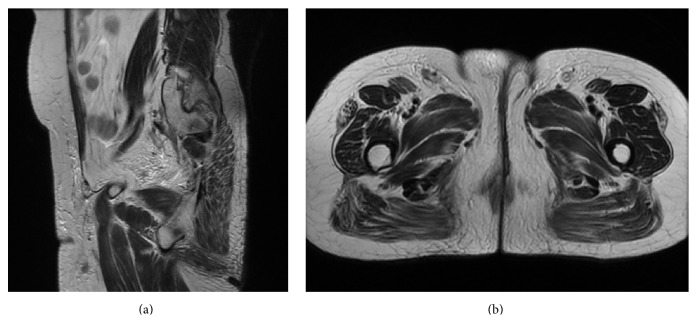
((a) and (b)) Posttreatment MR images in sagittal and axial section show complete resolution of the inguinal lymph nodes.

Magnetic resonance imaging of the pelvis demonstrated an irregular, intermediate signal intensity lesion in the left side of the labia of size of 10 mm × 10 mm along with multiple oval to round lymph nodal masses in the right superficial inguinal region measuring 5.3 cm × 7.5 cm in size (Figures 3(a) and 3(b)). Owing to the fixity of the contralateral inguinal nodes and the large size, neoadjuvant chemotherapy with Cisplatin of 100 mg/m^2^ and 5-Fluorouracil of 1000 mg/m^2^ was given for four cycles. Following this, the primary tumor and the contralateral inguinal nodes regressed completely (Figures 4(a) and 4(b)). After this, definitive chemoradiation was delivered with five cycles of weekly Cisplatin of 40 mg/m^2^ and external beam pelvic irradiation up to a dose of 59.4 Gy in 33 fractions. After a dose of 50.4 Gy to the whole pelvis, a boost dose of 9 Gy to the primary tumor site and the inguinal lymph nodal site along with a 2 cm margin was delivered. Patient has been followed up regularly and is disease-free 11 months after treatment.

## 3. Discussion

We describe a rare case of SCACP in vulva with locoregional metastases. A total of less than 30 cases of SCACP have been described in literature till date [7, 8]. The average age of diagnosis is 66 years [11]. Although, it most commonly occurs in the head and neck region, it may occur in other sites like perianal region, suprapubic region, calf, and arms [9]. Clinically, the lesion is long-standing, skin-coloured or yellowish, and papular in nature, which suddenly increases in size along with bleeding or ulceration [12].

The histopathological diagnosis of SCACP is sometimes difficult and may resemble hidradenocarcinoma papilliferum, apocrine ductal adenocarcinoma, and cutaneous metastases of malignancies microscopically [13]. While hidradenocarcinoma papilliferum and apocrine ductal adenocarcinoma are dermal tumors and do not show any epidermal changes, SCACP often expresses p63, which confirms its sweat gland origin, rather than a cutaneous metastasis [14, 15].

SCACP is generally a low-grade malignancy, with only two cases of recurrence being reported [7, 9]. Locoregional lymphatic metastases have been reported in only four patients to date [1, 7, 8], with none of them having any distant metastases [13, 14].

Due to the infrequent occurrence of the tumor, no standard treatment protocol has been advocated [8, 14]. Wide local excision has been advocated most commonly [8]. Sentinel lymph node biopsy has been performed along with excision in some cases [8, 14]. Mohs micrographic surgery has also been performed by Chi et al. [16] in a SCACP of the right auricle, with good disease control 6 years postoperatively. The role of chemotherapy or/and radiotherapy to SCACP is not clear. Adjuvant radiation therapy was delivered in one patient described by Arslan et al. [7], which then developed locoregional metastases.

In the current report, the patient had SCACP with locoregional failure postoperatively, which responded to chemotherapy followed by chemoradiation and is without any recurrence or distant metastases for 11 months after treatment.

## 4. Conclusion

SCACP is an extremely rare adnexal tumor which may occur in unusual sites like vulva. Locoregional lymphatic metastases, though infrequent, should always be investigated for. Though, as of now, wide local excision is the standard treatment, chemotherapy and radiation therapy can also achieve a good tumor control in cases of inoperable SCACP or in patients declining surgery. The role of chemoradiation has to be further investigated, especially in cases of locoregional disease spread. Regular follow-up is utterly important to detect any locoregional failure or recurrence early, as aggressive treatment may achieve good tumor control.

## References

[1] Numata M., Hosoe S., Itoh N., Munakata Y., Hayashi S., Maruyama Y. (1985). Syringadenocarcinoma papilliferum. *Journal of Cutaneous Pathology*.

[2] Woestenborghs H., Van Eyken P., Dans A. (2006). Syringocystadenocarcinoma papilliferum in situ with pagetoid spread: a case report. *Histopathology*.

[3] Helwig E. B., Hackney V. C. (1955). Syringocystadenoma papilliferum: lesions with and without naevus sebaceous and basal cell carcinoma. *Archives of Dermatology*.

[4] Niizuma K. (1976). Syringocystadenoma papilliferum: light and electron microscopic studies. *Acta Dermato-Venereologica*.

[5] Hoekzema R., Leenarts M. F. E., Nijhuis E. W. P. (2011). Syringocystadenocarcinoma papilliferum in a linear nevus verrucosus. *Journal of Cutaneous Pathology*.

[6] Dissanayake R. V. P., Salm R. (1980). Sweat-gland carcinomas: prognosis related to histological type. *Histopathology*.

[7] Arslan H., Diyarbakrl M., Batur Ş., Demirkesen C. (2013). Syringocystadenocarcinoma papilliferum with squamous cell carcinoma differentiation and with locoregional metastasis. *Journal of Craniofacial Surgery*.

[8] Satter E., Grady D., Schlocker C. T. (2014). Syringocystadenocarcinoma papilliferum with locoregional metastases. *Dermatology Online Journal*.

[9] Leeborg N., Thompson M., Rossmiller S., Gross N., White C., Gatter K. (2010). Diagnostic pitfalls in syringocystadenocarcinoma papilliferum: case report and review of the literature. *Archives of Pathology and Laboratory Medicine*.

[10] Abrari A., Mukherjee U. (2011). Syringocystadenocarcinoma papilliferum at unusual site: inherent lesional histologic polymorphism is the pathognomon. *BMJ Case Reports*.

[11] Peterson J., Tefft K., Blackmon J., Rajpara A., Fraga G. (2013). Syringocystadenocarcinoma papilliferum: a rare tumor with a favorable prognosis. *Dermatology Online Journal*.

[12] Park S. H., Shin Y. M., Shin D. H., Choi J. S., Kim K. H. (2007). Syringocystadenocarcinoma papilliferum: a case report. *Journal of Korean Medical Science*.

[13] Requena L., Kiryu H., Ackerman A. B. (1998). Ackerman's histologic diagnosis of neoplastic skin disease: a method by pattern analysis. *Neoplasms with Apocrine Differentiation*.

[14] Aydin O. E., Sahin B., Ozkan H. S., Gore O. (2011). A rare tumor: syringocystadenocarcinoma papilliferum. *Dermatologic Surgery*.

[15] Paradiso B., Bianchini E., Cifelli P., Cavazzini L., Lanza G. (2014). A new case of syringocystadenocarcinoma papilliferum: a rare pathology for a wide-ranging comprehension. *Case Reports in Medicine*.

[16] Chi C.-C., Tsai R.-Y., Wang S.-H. (2004). Syringocystadenocarcinoma papilliferum: successfully treated with Mohs micrographic surgery. *Dermatologic Surgery*.

